# P-2062. Hepatitis B Serostatus in the New York City Jail System, 2019–2023

**DOI:** 10.1093/ofid/ofaf695.2226

**Published:** 2026-01-11

**Authors:** Batya Koenigsberg, Raphael Simonson, Natalia Gonzalez Varela, Malia Mackey, Chibuzo U Enemchukwu

**Affiliations:** New York City Health+Hospitals Correctional Health Services, New York, New York; New York City Health+Hospitals Correctional Health Services, New York, New York; New York City Health+Hospitals Correctional Health Services, New York, New York; New York City Health+Hospitals Correctional Health Services, New York, New York; New York City Health+Hospitals Correctional Health Services, New York, New York

## Abstract

**Background:**

Prevalence estimates of hepatitis B virus (HBV) infection, immunity, and vaccine eligibility in jails vary widely and are not well studied. Jails house individuals from marginalized communities who face high burdens of comorbid conditions, including substance use disorders, HIV, and hepatitis C (HCV), which all share risk factors for HBV. Assessing HBV serostatus in a large urban jail provides critical insights to effective screening and prevention initiatives. This study describes HBV serostatus among persons experiencing incarceration in the New York City (NYC) jail system.

**Methods:**

This retrospective study includes all persons in the NYC jail system between October 2019 and December 2023 with HBV serologic testing in our system. Past HBV infection was defined as having HBV core (anti-HBc) antibody, and current HBV infection as surface antigen (HBsAg) positivity. HBV immunity was defined as having antibody to HBV surface antigen (anti-HBs), and HBV immunity by vaccination as having anti-HBs with no anti-HBc and no HBsAg. Select demographics and medical history were also examined and described.

**Results:**

2282 persons were included, 268 (11.7%) were women and 2014 (88.3%) were men. In all, 619 (27.1%) had serologic evidence of past HBV infection. 91 (4.0%) had evidence of current infection. 1310 (57.4%) were immune, of whom 1002 (76.5%) were immune by vaccination. 645 (28.3%) persons had no evidence of past or current HBV infection or immunity and were susceptible to HBV. Among those susceptible to HBV, 81 (12.6%) were women, 143 (22.2%) were living with HIV, 183 (28.4%) had laboratory evidence of HCV exposure, and 429 (66.5%) had a history of select substance use disorders.

**Conclusion:**

The majority of individuals in our cohort were immune to HBV (57.4%), with nearly three-quarters of those immune by vaccination. 4% of the cohort had serological testing indicating current infection. However, a much larger proportion (28.3%) was susceptible to HBV and vaccine-eligible. We found that a substantial portion of individuals eligible for vaccination had medical conditions associated with HBV risk. These findings underscore the importance of ongoing prioritization of HBV screening and prevention protocols in jail settings.

**Disclosures:**

All Authors: No reported disclosures

